# Enhancing products performance evaluation through hybrid DistilRoBERTa and BiGRU models

**DOI:** 10.1371/journal.pone.0348316

**Published:** 2026-05-26

**Authors:** Shoukat Ullah, Aurangzeb Khan, Aman Ullah, Muhammad Nawaz Khan, Rehan Tariq Chohan, Farhan Aadil, Khair Ullah Khan

**Affiliations:** 1 University of Science & Technology, Bannu, Pakistan,‌‌; 2 Department of Commerce Education and Management Sciences, Higher Education, Archives and Libraries Department, Khyber Pakhtunkhwa, Peshawar, Pakistan,‌‌; 3 Department of Smart Security, Gachon University, Seongnam, Republic of Korea; 4 Faculty of Modern and Technological Sciences, The National University of Malaysia, Qatar (UKM-Qatar Campus), Qatar; 5 Computer Engineering Department, Sivas University of Science & Technology, Sivas, Türkiye; Gachon University, KOREA, REPUBLIC OF

## Abstract

Online reviews have a direct bearing on what prospective customers will choose to buy. Consumers need help to make effective use of online reviews for purchasing decisions. The current sentiment analysis methods often overlook the complexity and usability of products in reviews which are critical for potential buyers. The existing approaches solely rely on sentiment polarity but this approach offers a comprehensive way to analyze product’ reviews by integrating appraisal theory with hybrid deep learning methods. This research study aims to explore product complexity in customer reviews by utilizing transformer and recurrent neural network-based models. Amazon product reviews dataset is manually annotated according to perceived complexity using attitudinal categories of appraisal theory, i.e., appreciation and judgment and then cross-validated through shapley additive explanations method of eXplainable AI. Appreciation has to do with how much a product is user-friendly or has a learning curve to operate. Judgment deals with evaluation of products on the basis of effectiveness and appropriateness. The proposed model uses a hybrid approach by combining DistilRoBERTa, a pre-trained transformer model, and a bi-directional gated recurrent unit, a recurrent neural network-based model, to learn both contextual and sequential dependencies in product reviews. The proposed approach makes use of fine-tuned DistilRoBERTa embeddings for extracting features, and this model is improved further using a bi-directional gated recurrent unit layer, which takes into account the context of the past and the future. A 5-fold stratified cross-validation method is used to address imbalance learning, with class weighting applied to further balance the impact of sentiment classes in training. The proposed model has achieved a mean fold accuracy of 96.13%,which is higher than that of existing state-of-the-art approaches such as Random Forest (89.93%) [11], DistilBERT with advanced embeddings (92%) [25], XLNet (89.62%) [28], and GPT-based sentiment models (94.5%) [30]. By utilizing Shapley Additive Explanations (SHAP) for explainability, this model provides transparency in understanding emotional tendencies, functional effectiveness, and overall user perceptions, offering insights that traditional models lack. This framework provides a scalable, automated solution for Amazon products’ performance evaluation and provides insights into emotional tendencies, functional effectiveness and overall perceptions from the users. It will help in optimizing product development strategies using advanced natural language processing techniques with explainability by setting a new standard for understanding products’product feedback.

## Introduction

Online reviews about the products have become a pivotal source of information for customers that helps them make informed decisions. Sentiment analysis (SA) plays a key role in the automatic extraction of opinions from the reviews. Through processing the huge scales of reviews, SA helps businesses to get an idea about people’s satisfaction, leading to better offerings. This process leads to a higher level of consumer trust and decision-making by giving more understanding of public sentiment [1]. SA also plays an essential role in the e-commerce platforms, where reviews and comments provide critical business intelligence [2]. In a market-driven design paradigm, an understanding of user experience is very important because it can be a source of information, such as user preferences, choices, and other features. Traditional methods such as questionnaires, surveys, and self-reports are typically used to gain insight during user experience studies [3]. SA has various limitations, which make it difficult to understand what people are saying and their polarities. These obstacles make it incredibly difficult to manage to extract exact sentiments from the text. The process entails the use of natural language processing (NLP) and text mining to identify the subjective information. Issues such as ambiguity, sarcasm, and language that is dependent on context make analysis difficult. Additionally, language and expression differences can be a source of misinterpretations [4]. The conventional SA has been advanced by the appraisal theory. The authors in [5] predicted perceived product quality via the attitude system of the appraisal framework and employed the principal element, i.e., appreciation of the same system. In today’s world, enhancing users’ liking by analyzing reviews in the e-commerce world is becoming more important. However, it is hard to predict the sentiment in these reviews accurately because of factors such as variable review sizes, word order, and the complex logic behind the contents [6]. Traditional SA techniques, which basically involve determining the positive or negative feedback, do not capture the complexity of human sentiment. They tend to ignore finer areas of consumer experience, like usability of the products, effectiveness, and emotional engagement. In this research framework, we proposed a more advanced deep learning (DL)-based approach to SA by means of incorporating some more complex linguistic markers and providing a holistic evaluation approach of the performance of Amazon products. At the center of this research is a hybrid model of DL that consists of the pre-trained transformer-based DistilRoBERTa and the bi-directional gated recurrent unit (BiGRU) layer. DistilRoBERTa, which is known to be an efficient model in natural language understanding natural language understanding (NLU) tasks, fills up the context information that is embedded in reviews. Meanwhile, the BiGRU layer gives support to sequential dependencies modeling, which sees the feeling expressed in a review as felt from past and future feedback. This hybrid architecture can be considered an approach to thinking outside the simple identification of polarity and looks towards the analysis of more complex linguistic phenomena, such as the complexity of products and usability, to make a richer understanding of consumer sentiment. A unique contribution of this research is the theory behind the research, which is based on appraisal theory. Unlike traditional SA, which classifies feedback in simple positive or negative sentiment, appraisal theory allows us to break down user reviews in appreciation and judgment dimensions. These dimensions provide a better understanding of the emotional involvement, aesthetic value, and functionality efficiency of the product and provide a multi-dimensional picture of the experience for the consumer. This not only enables businesses to increase the depth of their knowledge of sentiment but can also allow them to analyze the reasons behind the user’s satisfaction or dissatisfaction, for example, the perceived complexity of the product or its usability in real-world scenarios. Another reason for this research is that they consider the use of eXplainable AI (XAI). In real-world applications, particularly when analyzing consumer feedback, it is critical for businesses to be able to understand the thought process of the model. This research is using shapley additive explanations (SHAP) to validate the accuracy of the manually annotated labels.

Unlike traditional SA models that focus on a simple polarity classification (0 or 1), our approach presents a multi-dimensional framework to discuss reviews by focusing on an Appraisal theory, which splits appraisal into two dimensions: appreciation and judgment. This makes it a more nuanced understanding of customer feedback to capture the complexity and usability of products, which is often overlooked in other models. Furthermore, by combining two AI models, DistilRoBERTa and BiGRU, our proposed model is able to capture the contextual and the sequential dependencies in the product reviews, which improves the quality of our analysis of product reviews. In addition to this, we also used SHAP to validate the manual annotations and to ensure accuracy and transparency in the model’s predictions. SHAP helps us understand the factors that influence the sentiment classification and gives us information about the emotional and functional aspects of the reviews, which is of great importance to improve the interpretability of the model. By addressing these aspects, the proposed approach is a significant improvement over the state-of-the-art SA methods, providing a deeper understanding of customer sentiments as well as analyzing product reviews more accurately and comprehensively than the existing state-of-the-art models.

### Main contributions of the study

The major contributions of this work are as follows.

- To develop a multi-dimensional SA model based on the appraisal theory to decompose reviews into appreciation and judgment dimensions to overcome the classic positive/negative classification.
- To manually annotate the reviews to capture the deeper linguistic phenomena and provide a richer understanding of the feeling.
- To use the SHAP for validation of annotated reviews, particularly the accuracy and transparency of predictions of the proposed model.
- To present the hybrid model, which is the combination of DistilRoBERTa and BiGRU, to enhance the SA by taking both context and sequential dependencies into consideration in reviews.
- To validate and improve the reliability of the proposed model using the K-fold stratified cross-validation method.

## Literature review

SA and OM are commonly used fields to analyze textual data from various sources, such as social media and Instagram. These fields enable businesses to gain an in-depth insight into buyers’ feedback about their products [7]. SA is a computational research in drawing the subjective information from the text [8]. In [9], the authors proposed the application of traditional feature extraction methods like bag of words (BoW), TF-IDF in SA tasks to classify the text accurately. In addition, the authors also proposed a hybrid combination of TF-IDF and BERT in product reviews classification. Furthermore, the authors added negation handling within these hybrid models, resulting in an 88% accuracy in evaluation as compared to existing methods. The authors in [10] suggested the SA for the product reviews across various e-commerce platforms, including Amazon, Flipkart using recurrent neural networks (RNN). The convolutional neural networks (CNN) based LSTM model for Amazon reviews has also been introduced, which achieved a higher accuracy of 94%, in comparison to the previous models. M. Chugh et al. [11] highlighted the issues of sequence length and textual order in the analysis of sentiments in user reviews on e-commerce websites by using TextBlob. The process consisted of data collection and cleaning, feature extraction, and sentiment classification using random forest (RF). The accuracy of the proposed model was 89.93%. In [12], the authors introduced a prediction pipeline for the detection of aspects and SA based on pre-trained models, including BERT and T5. These models were trained using synthetic and labelled datasets to classify customer reviews of eco-friendly products in positive, negative, and neutral sentiments. BERT outperformed T5 by gaining an accuracy of 92%.

The authors in [13] proposed the sigTan-Beta Activation Function for CNN to enhance SA of Amazon product reviews and resolved the challenges such as phrase length, text order variations, and logical complexities in the reviews. The approach consisted of preprocessing, text to vector by using Word2Vec, and feature extraction using CNN. The performance obtained by the use of sigTan-Beta Activation Function had better accuracy as compared to the ABO-RF algorithm. The authors in [14] employed a BERT model to categorize the reviews of Amazon Electronics products into positive, negative, or neutral. The model was trained using a dataset that contains 6,739,590 reviews, whereby approximately 36,000 reviews per sentiment category were used. The model attained an accuracy of 93.5% and an F1 score of 93.5%, which showed its reliability and scalability in classifying complex reviews. In [15], the authors proposed SA of Amazon product reviews in communication technology in a four-dataset approach. They made an improvement in the proposed model by adding an extra step of the title of the product and headlines for context. Using TF-IDF, Word2Vec, and FastText embeddings, FastText performed the best when combined with the XGBoost and CatBoost algorithms to create the FastXCatStack model, which had an accuracy score of between 93% and 94%. Linear support vector machine (SVM) using FastText gave it 91% accuracy on software reviews. Arif, et al. [16] examined customer interactions with Amazon’s “@AmazonHelp” to predict sentiment changes. Using the Twitter API, they extracted and pre-processed the English language tweets and categorised the conversations for analysis. Different machine learning (ML) algorithms, including K-nearest neighbor (KNN) and SVM, were tested. The results of bagging classifier with RepTree were the highest with an accuracy rate of 82% compared to other approaches.

The authors in [17] conducted SA on the Amazon musical instrument reviews. Preprocessing of data, exploratory data analysis, and labeling the products based on customer ratings, i.e., positive for ratings is greater than 3.0, neutral for ratings is equal 3.0, otherwise negative was performed. After the feature engineering and resampling using SMOTE, the logistic regression (LR) proved to be the best model among others, with an accuracy of 94.80% after tuning the hyper-parameters. In [18], the authors performed SA on e-commerce reviews by testing LSTM and CNN-LSTM models. The proposed system used real-time data from Amazon reviews about the cameras, laptops, mobile phones, and other products, and applied preprocessing techniques such as stopwords removal and tokenization. The LSTM model had achieved higher accuracy in comparison to CNN-LSTM model. In [19], the authors proposed a sentiment classification model, named LeBERT, by integrating sentimental lexicon, BERT and CNN to overcome the limitations of the existing text representation techniques. CNN was used for feature mapping. The effectiveness of the model was tested with three public data sets, Amazon, IMDB, and Yelp reviews, and the proposed model achieved a score of 88.73% in F-measure, which was better than the performance of existing models for binary sentiment classification. In [20], the authors suggested using SA on Amazon customer reviews to determine product and retailer quality. They used term-based methods and n-gram, data preprocessing, and feature extraction to classify the reviews as positive, negative, and neutral reviews. The SVM with unigram gave the highest accuracy of 82.27% with 82% of precision, 80% of recall, and an F1 score of 72%. In [21], the authors performed SA from Amazon mobile phone reviews using a combination of each of the RNN variants (LRNN, GLRNN, GRNN, UGRNN) along with embeddings (GloVe, Word2Vec, and FastText) and presented the results of the combinations using standard metrics. According to their study, the best accuracy (93.75%) on the unbalanced data was found for GLRNN with FastText, whereas the best accuracy (88.39%) on the balanced data was found for LRNN. The authors in [22] proposed an information processing model, Pearson correlation coefficient-based Harris Hawks Optimization (HHO) RNN-LSTM model (PCCHHO-RNN-LSTM), for sentiment classification. The approach reduces feature dimensionality by using the correlation analysis and selects non-redundant features using HHO. Experiments on reviews from Amazon were 95.8% accurate, better than existing methods.

In [23], the authors proposed a new naive Bayes (NB) based SA framework that can be used for the classification of online product reviews. Chi-square feature selection (top-K = 450) and TF-IDF weighting were taken to tackle the feature sensitivity. Experiments were performed on the Amazon labelled dataset from UCI, consisting of 1000 reviews. This proposed model has improved the accuracy from 82% to 83% as compared to standard NB. The authors in [24] presented a comparative study of an SA study of Amazon multi-domain product reviews. Reviews were classified as positive or negative using three different model SVM, CNN, and BERT. Experimental results proved that BERT is more accurate than CNN and SVM with an accuracy of 95.34%. The authors in [25] compared SA models, based on ML, DL, and transformer, using the Amazon reviews, evaluating various methods of word embedding using standard metrics. FastText performed the best in traditional and LSTM models, where the DistilBERT model had the highest accuracy rate of 92%. In [26], the authors performed SA based experimental research on the customer reviews of various platforms such as twitter, IMDb, Yelp and Amazon considering the challenges in accuracy and context based predictions. They proposed fine-tuned BERT model and compared the performance with Linear SVM custom, FastText, BiLSTM and hybrid (FastText-BiLSTM). The evaluation proved the fine-tuned BERT to be better than other models and was able to predict the sentiment better by achieving an accuracy of 90%.

Shobayo, Olamilekan, et al. [27] proposed Google’s Pathways Language Model (GooglePaLM) for SA on Amazon fashion reviews, and compared it with VADER and BERT. The study found that big language models are more efficient than conventional rule-based systems for handling complicated linguistic features in customer feedback. Danyal, Mian Muhammad, et al. [28] evaluated XLNet for sentiment classification and longformer encoder-decoder (LED) for summarization of user reviews. XLNet had a high accuracy score (89.62%) on Amazon Fine Food reviews dataset that was able to capture very nuanced sentiments and LED produced concise and coherent summaries with the highest score in ROUGE, METEOR, MoverScore and BERTScore-F1 metric. BERT, RoBERTa, and BART models also performed well for SA and outperformed other traditional ML models and sequence-to-sequence model T5. The authors in [29] examined SA models on reviews of books from Amazon using traditional ML techniques (NB, KNN, CART), LSTM and the transformer-based RoBERTa. RoBERTa was superior to all models with an accuracy score of 96.30%, which proved it to be strong with complex and semantically rich text. Traditional models had limitations in describing subtle textual relationships and LSTM also had problems of scalability and overfitting. In [30], the authors examined the performance of transformer models including DistilBERT, ELECTRA, T5, ERNIE and GPT-2 on the task of SA of Amazon product reviews. Performance was compared using metrics such as accuracy, precision, recall and F1-score where the results were benchmarked against the Lexicon-Enhanced BERT model. GPT-2 scored the highest accuracy of 94.5% which shows good capacity to capture and analyse sentiments.

The authors in [31] performed SA on Amazon reviews by combing DL models with both context-independent (Word2Vec, FastText) and transformer-based embeddings. Performance was measured using measures on various models and feature extraction methods. BERT got its highest accuracy score that reached 87.13% with GRU, and ALBERT performed best with BiGRU with 87.1%. In [32], the authors analyzed SA on Amazon products reviews using a different ML models. The RNN was able to provide an accuracy of 85% when classifying customer sentiments. The authors in [33] suggested a hybrid GPT-based model that uses DistilBERT to extract features, BiLSTM to classify the sentiment, and XGBoost to forecast regional trends for the SA of Amazon product reviews. The accuracy of the proposed approach was 92.3% and 87.6% of positive and negative insights, respectively, and F1 scores of 90.5% and 84.9%, showing balanced precision and recall, respectively. The authors in [34] proposed a social media marketing approach based on an attention-based RNN for a four-class sentiment classification for Amazon product categories. The proposed model proved better performance from Amazon data by achieving an accuracy of 85%. In [35], the authors proposed a product feature improvement model (CESC) based on consumer online reviews, which combined the word segmentation, LSTM neural networks, and LDA topic models. This model works to extract product features and sentiments from the reviews, so the companies can improve the features of the product based on the feedback from the consumers. The authors in [36] compared different traditional ML methods, and among them NB model got superior performance.

In [37], the authors examined IoT and ML to make better decisions in e-commerce and customer experiences. From various ML techniques, AdaBoosting was compared to other models such as DL models and found to be better with an accuracy of 88%, and an F1 score of 92%. This study showed how a combination of IoT and ML can involve the efficient use of product recommendations and demand forecasting for more effective and customer-centric retail strategies. In [38], the authors proposed a recommendation system that was based on collaborative filtering in combination with SA based on a fine-tuned BERT model, which resulted in 91% accuracy. A. Romadhony, et al. [39] compared conventional ML models (SVM, MNB) and DL models (LSTM, BiLSTM) with a large Indonesian product review dataset. BiLSTM gave the best accuracy of 79% for sentiment classification and 57% rating prediction. A. Perti, et al. [40] applied the SA approach to classify product-related tweets as either positive or negative. They utilized CNNs along with LSTMs and an ensemble approach of CNN models. The ensemble approach achieved 84.95% of accuracy. The method was based on n-gram-based word embeddings and outperforms various existing methods.

The authors [41] performed SA on Samsung Amazon reviews through exploratory data analysis. NB, LR, and RF classifiers were employed. RF model gained the highest accuracy of 90%. In [42], the authors came up with a novel method of performing SA that effectively uses emojis and emoticons in customer feedback, especially on the US Airline tweet dataset. Using these elements, the BEERT model (variant III) achieved 92% accuracy, which was 9% higher than the accuracy of frameworks currently available on the market. Amanullah et al. [43] proposed a DistilBERT-based model to predict the perceived quality of mobile apps. The model achieved 92.88% of accuracy, which was better than the baseline strategies. In [44], the authors proposed an SA-based model, namely LSIBA-ENN, using online product reviews. Using the LTF-MICF method, LSIBA-ENN achieved a recall of 87.79%, which was better than other classifiers. In [45], the authors presented a model based on review summarization using NLP and an LSTM algorithm to analyze the sentiments and give insights into consumer behavior. The authors in [46] suggested a hybrid model that integrates Transformer-based DeBERTa and DL based IDCNN for effective aspect-level feature extraction, and used an attention-based BiLSTM-CRF model for sentiment classification in customer reviews. Experimental results on four benchmark datasets showed that the model outperformed existing methods, achieving accuracy scores up to 93.08%. M. Kuppusamy and A. Selvaraj [47] proposed a novel hybrid CNN model, combining BiLSTM and CNN to process user feedback for SA. The detailed comparison of SA techniques as discussed above is shown in Table 1.

**Table 1:** Comparison of Sentiment Analysis Techniques and Outcomes.

Set A				Set B			
Ref	Technique	Outcome	Acc.	Ref	Technique	Outcome	Acc.
[9]	BoW, TF-IDF	Accurate classification	–	[28]	XLNet, LED	Sentiment + summarization	89.62%
[10]	RNN-based SA	E-commerce sentiment	–	[29]	RoBERTa	Amazon book reviews	96.30%
[11]	TextBlob + RF	Order issues resolved	89.93%	[30]	DistilBERT, GPT-2	Amazon SA	94.5%
[12]	BERT, T5	Review classification	92%	[31]	CNN, GRU, BERT	Transformer embeddings	87.13%
[13]	CNN + sigTan-Beta	Logical complexity handled	94.5%	[32]	RNN-based SA	Customer reviews	85%
[14]	BERT	Multi-class reviews	93.5%	[33]	GPT + BiLSTM	Trend forecasting	92.3%
[15]	TF-IDF, XGBoost	Feature extraction	93–94%	[34]	Attention RNN	Multi-class SA	85%
[16]	KNN, SVM	Twitter sentiment	82%	[35]	LSTM, LDA	Feature analysis	89.52%
[17]	SMOTE + LR	Musical reviews SA	94.8%	[36]	NB, SVM, NN	Amazon reviews	94%
[18]	LSTM, CNN-LSTM	Real-time reviews	94%	[37]	IoT + ML	Experience improvement	88% accuracy, F1-score 92.7%
[19]	Lexicon, CNN, BERT	Representation limits solved	88.73%	[38]	BERT + CF	Recommendation SA	91%
[20]	SVM n-grams	Amazon SA	82.27%	[39]	BiLSTM	Large-scale reviews	79%
[21]	RNN + embeddings	Balanced SA	93.75%	[40]	CNN, LSTM ensemble	Tweet classification	84.95%
[22]	RNN-LSTM + HHO	Dimensionality reduction	95.8%	[41]	RF, NB, LR	Samsung reviews	90%
[23]	NB + TF-IDF	Product reviews	83%	[42]	Emoji-aware SA	Airline tweets	92%
[24]	SVM, CNN, BERT	Multi-domain reviews	95.34%	[43]	DistilBERT	App quality prediction	92.88%
[25]	FastText, DistilBERT	Amazon SA	92%	[44]	LSIBA-ENN	Recall improvement	87.79%
[26]	Fine-tuned BERT	Customer reviews	90%	[45]	NLP + LSTM	Review summarization	92.81%
[27]	GooglePaLM	Compared with VADER	93%	[46]	DeBERTa, IDCNN	Aspect SA	93.08%
				[47]	CNN + BiLSTM	User feedback SA	93.6%

Table notes: Comparison of major sentiment analysis techniques, their application context, and reported performance across datasets.

Despite the advancements in SA, most of the existing models are mainly dedicated to analyzing the binary sentiment polarity (0 or 1), while overlooking the multidimensionality of customer feedback. Although transformer-based models have shown great promise, such models are prone to neglecting the sequential dependencies in a product review, which can result in lower accuracy in sentiment prediction. Many current methods, especially in black box models, have the shortcoming that they are not explainable in their predictions. This opacity in understanding the underlying factors that affect sentiment prediction makes the model less practical for practitioners, who may not have the time or the desire to examine it more closely, making it difficult to use in areas such as e-commerce where interpretability is critical. This research, therefore, seeks to resolve these problems by introducing a new hybrid model that uses DistilRoBERTa and BiGRU to enable the model to capture both contextual and sequential dependencies in product reviews. Additionally, by using the Appraisal theory for deconstructing reviews into two dimensions of appreciation and judgment, we have a more nuanced understanding of customer sentiment. The utilization of SHAP in this study makes SA more transparent and accurate by performing a manual annotation hot check, providing better explanations for models and even better insights regarding the product complexity and usability. By combining these innovative ingredients, our approach allows important progress over the existing state-of-the-art methods in SA in terms of providing a deeper analysis of user reviews and transparency in the models which is a crucial aspect for real-world applications in e-commerce platforms such as Amazon.

### Problem statement

The exponential growth of online reviews by the masses, especially on Amazon, has made it difficult for businesses to manually analyze massive amounts of feedback, and SA has become a tool to gain meaningful insight. However, current SA techniques, after thoroughly studying the related work, emphasize mainly polarity classification but neglect complex linguistic features such as usability, complexity, or context, which are important to know users’ preferences. This research seeks to fill in the gaps by creating a hybrid DL model in the form of the combination of DistilRoBERTa and a BiGRU layer that will analyze Amazon product reviews with aspects of appraisal theory (appreciation, judgment) to analyze nuanced sentiments associated with product complexity and usability.

## Methodology

This section includes data acquisition, data annotation, text preprocessing, and a detailed description of the proposed model, as well as its pseudocode.

### Data acquisition

In this study the proposed approach is implemented in Google Colab using the Amazon Product Reviews dataset taken from [48] having 28332 reviews and 24 attributes, out of which 5373 reviews based on perceived complexity were selected.

### Data annotation and its guidelines

The annotation was done by three experienced annotators, all of which have a background in computer science and data science, combined with familiarity in SA and NLP tasks. The annotators were carefully chosen to be consistent and reliable in the comprehension of the components of the Appraisal theory and product complexity. The data annotation guidelines are created to ensure consistency across the annotation process, which contains specific instructions on how to categorize product reviews based on appreciation and judgment components of the Appraisal theory. Annotators are asked to rate the ease of use, simplicity of a product, and advanced features that might suggest a higher level of expertise needed. For instance, an easy-to-use product that is easy to operate, with no specialized knowledge, would come under the category ‘Simple’ under the dimensions of appreciation. Annotators were instructed to determine the appropriateness of the complexity of the product to the potential consumers. A product that is intuitive and appropriate for a broad audience would be classified as “Simple” while a product with advanced features aimed at professionals would come under the category of “Complex” in the judgment dimension. After the annotation process, the Cohen’s Kappa coefficient is calculated to measure the level of agreement between the annotators. The final Cohen’s Kappa score was found to be 0.87, which is an indication of a high level of agreement among the annotators. This score indicates how reliable the manual labeling process is, and it helps to ensure that the dataset annotations are consistent and robust and can be used to train the model. The Cohen’s Kappa score of 0.87 shows high levels of agreement between the annotators, which exceeds the generally accepted cutoff point for inter-rater reliability of annotation tasks. This score means that the dataset that is labeled is of high quality and that the labeling is trustworthy.

In the case of the manual labeling of product complexity, Appreciation component of appraisal theory is concerned with how a product is valued on the basis of the features it has; for instance, user-friendly products are appreciated for their ease of use and simplicity, while more advanced products are recognized for the provision of specialized or professional-grade features that require a higher level of expertise. The Judgment component measures whether or not the complexity of the product is acceptable for the consumer. For example, a product that is easy to use and that suits the needs of general consumers would be considered “simple,” whereas a product with high-level features, which could be used by professionals or experts, would be “complex.” The examples of data annotations based on appraisal theory are shown in Table 2.

**Table 2:** Review-Based Assessment of Usability and Complexity.

Review	Appreciation	Judgment	Complexity	Reason
Tablet is very user-friendly and suitable for casual browsing	User-friendly	Appropriate	Simple	Easy to use and suitable for general users
Camera offers professional features but needs expertise	Advanced	Suitable for professionals	Complex	Rich features but difficult for general users
App is simple to navigate and meets basic needs	User-friendly	Appropriate	Simple	Appreciated for simplicity and usability
Software provides advanced analytics but requires learning time	Advanced	Suitable for experts	Complex	Advanced tools require specialized knowledge
Device is ideal for beginners with quick setup	User-friendly	Appropriate	Simple	Quick setup and beginner-friendly
Product has many features but can overwhelm beginners	Advanced	Inappropriate for beginners	Complex	Feature-rich but difficult for new users

Table notes: Evaluation of user reviews highlighting usability, judgment, and perceived complexity of different products.

### Text preprocessing

According to the authors in [49], text preparation is the process of improving the quality of text in reviews. Common words (like “the,” “and”), punctuation, and special characters are eliminated, the text is divided into sections, and word forms are made simpler.

#### Tokenization.

In this step we have tokenized the reviews because in NLP, tokenization is essential because it transforms unstructured text into more easily analyzed units. Tokenization makes text manipulation easier by dividing it into smaller units, or tokens, which is crucial for activities like ML and SA.

#### Conversion to lowercase.

In this step, we have converted reviews into lowercase to ensure the consistent performance of ML model.

#### Removal of punctuations.

In this step, we have deleted punctuation symbols like commas and periods, enabling the focus to be on the words alone for ML model and making it easier to recognize and assess the content.

#### Removal of stopwords.

We have removed the stopwords like “a,” “a,” “an,” and “the” etc to improve sentiment classification. By eliminating terms that add little value, the feature selection becomes more efficient.

### Description of proposed model

**Fig 1:**
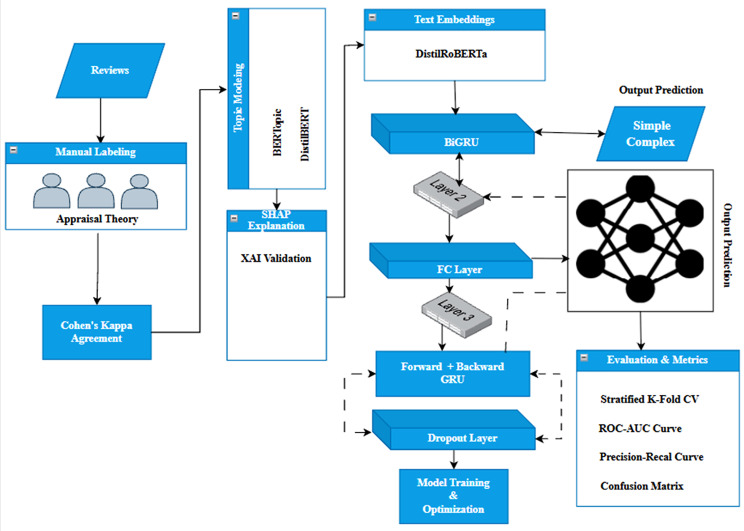
The Proposed Hybrid Model.

Our proposed model is based on RoBERTa as a lightweight and optimized version of the RoBERTa model and BiGRU layer to predict the perceived complexity of products. At first, is the manual labeling, in which the review is classified by the annotator as “Simple” or “Complex” depending on the perceived intricacies of the product. To try to quantify the inter-rater reliability of the manual labels, we use Cohen’s Kappa as a measure of the agreement between multiple annotators, which takes into account the chance of agreement and thus gives a much more robust measure of the consistency of labels. To aid validation of these manual labels, we add XAI techniques, in this case SHAP, to make sure that the model is transparent. The SHAP method ensures the semantic consistency of human-annotated labels and the model-based predictions to ensure that the reasoning process of the model is consistent with human expectations. Furthermore, the model includes handling negation in the preprocessing pipeline in order to deal with the syntactic complexity added by negation words (e.g., not complicated). The negation context is parsed for a correct complexity adjustment in order to ensure the semantic structure context determines the complexities that might get reversed by negations. In addition to manual labeling, topic modeling is used through BERTopic in order to obtain the latent semantic structure information in the text. CountVectorizer is used here to extract meaningful features from the text, which are modeled into topics that provide more information for the classification of complexity. Besides, SA is also done in a pre-trained model of DistilBert, which classifies the text sentiment as positive for Simple or negative for complex. The class of sentiment provides another level of analysis, as the tone of emotion can determine the perceived complexity of the given text, adding to the confirmation of categorization of complexity. The basic structure model takes advantage of the context embeddings of the DistilRoBERTa model, which uses the RobertaTokenizer to tokenize the text of the reviews and then passes them through the DistilBertModel. This results in high-dimensional embeddings, which represent semantic relations and contextual nuances between words. These embeddings are then fed to a BiGRU layer, which uses the bidirectional information propagation to capture the past and future dependencies from the input sequence, which helps the model to improve the ability to understand the syntactic structure and the long-range dependencies. The results from the BiGRU layer is passed through FC layer, which outputs the final predictions as positive and negative for the simplicity and complexity of products, respectively. In order to make sure that the generalization of the model, we add dropout regularization in the output of the BiGRU layer; this helps to avoid overfitting by randomly dropping the connections in the model during the training process. The model is trained with the help of cross-entropy loss and implemented using the AdamW optimizer, which uses weight decay, which helps in reducing the overfitting issue by penalizing the large weights so that it helps in improving the generalization of the results. Learning rate scheduling, used in this case with cosine annealing with warm restarts, is used to vary the rate of learning during the training in order to train the model in an efficient way and escape the local minima. Class imbalance is mitigated by incorporating class weighting in the loss function, which is calculated using the $compute_{c}lass_{w}eight$ function of scikit-learn, thus making sure that the model pays more attention towards under-represented classes while training. The model is evaluated using stratified K-fold cross-validation, which splits the dataset into k folds while preserving the distribution of classes. Thus, rather than just providing the evaluation of the model in a simple manner, the stratified K-Fold cross-validation makes sure that the evaluation is done in a fair and balanced way. The performance of the model is checked on the basis of accuracy, ROC AUC, precision, recall, and the confusion matrix. Through the combination of manual data annotation validation, XAI methods, and the DistilRoBERTa-BiGRU approach, our proposed model guarantees the success of not only high-accuracy text classification but also interpretable and transparent text classification predictions. The proposed model tuning strategies, like dropout regularization, learning rate scheduling, and class weighting helps in optimizing the performance of the model, avoiding overfitting and class imbalance. This combination of enhanced techniques of model tuning, XAI, and semantic processing creates better semantic coherence between machine prediction and manual labels and provides an end-to-end proposed framework for validating and changing complexity labeling processes in order to guarantee robust model performance. A diagrammatic representation of the proposed model is shown as Fig 1 and its pseudocode as Algorithm 1.

**Algorithm 1 Proposed Text Complexity Classification Model**

1:  **Input:** Raw textual dataset *D*

2:  **Output:** Classified labels {*Simple*, *Complex*} and evaluation metrics

3:  **Step 1: Manual Labeling and Negation Handling**

4:  Manually label dataset samples as *Simple* or *Complex*

5:  Compute inter-rater agreement using Cohen’s Kappa score

6:  Identify negation terms (e.g., “not”) preceding complex terms

7:  Adjust contextual interpretation of affected terms

8:  **Step 2: Topic Modeling**

9:  Apply BERTTopic with CountVectorizer to discover latent topics

10:  Perform sentiment analysis using DistilBERT

11:  Extract semantic topics and contextual features from text

12:  **Step 3: Model Definition and Layer Setup**

13:  Initialize DistilRoBERTa for contextual text embeddings

14:  Pass embeddings into Bidirectional GRU (BiGRU)

15:   Forward GRU for sequential encoding

16:   Backward GRU for reverse contextual encoding

17:  Apply Dropout layer for regularization

18:  Use Fully Connected (FC) layer for classification output

19:  **Step 4: Model Training and Optimization**

20:  Define Cross-Entropy loss function

21:  Initialize AdamW optimizer for parameter updates

22:  Apply Cosine Annealing learning rate scheduler

23:  Incorporate class weighting to handle imbalanced data

24:  **Step 5: Model Prediction**

25:  **for** each input sample *x*_*i*_ **do**

26:   Pass *x*_*i*_ through the trained model

27:   Predict label $l_{i}\in \{Simple,Complex\}$

28:  **end for**

29:  Store predicted labels

30:  **Step 6: Model Evaluation**

31:  Perform Stratified K-Fold Cross-Validation

32:  Compute evaluation metrics:

33:   Accuracy, Precision, Recall, and F1-score

34:  Generate ROC-AUC curve

35:  Generate Precision-Recall curve

36:  Construct Confusion Matrix

## Experimental setup for model training and evaluation

The various libraries that are imported are PyTorch for training our model and tensor management, Transformers for pre-trained models such as DistilRoBERTa and BERT, Pandas for data manipulation, Sklearn for model evaluation, cross-validation, and class balancing, and Matplotlib/Seaborn for visualising results such as the confusion matrix and performance curve. Google Colab is allowing access to Google Drive for easy data attainment. The data set is then loaded from the CSV file, and some file preprocessing (removal of missing values and conversion of sentiment labels into binary values). NLTK is used to increase the complexity of keywords as well as negation. A custom Dataset class has been written to do the tokenization using DistilRoBERTa, in order to ensure that compatible with inputs to the model. Model architecture is DistilRoBERTa embeddings, BiGRU Layers (bidirectional dependency), and fully connected layers for the sentiment classification with dropout regularization to avoid overfitting. Cross-validation is used with stratifiedkfold and the optimizers AdamW and CosineAnnealingLR for dynamic learning rate adjustment is used. During the process of training, data is passed in batches and behaviors such as loss, correctness, precision, recall, and F1 score are monitored. The model is tested using the ROC curve, the Precision-Recall Curve, and finally, a confusion matrix is plotted to perform a classification performance analysis. For the SA, BERTopic for topic modelling and SHAP for explaining model prediction are used. Performance metrics, including accuracy, precision, recall, F1 score, and ROC-AUC, are calculated to check how well, the model is working, which are complemented by performance curves and confusion matrices providing additional information about the class imbalance.

### Evaluation metrics

Evaluation metrics are used to assess the model’s effectiveness. The details of various evaluation metrics,s such as accuracy, F1-score, precision, recall, and F1-score, are as follows.

**Accuracy:** Accuracy measures the percentage of accurately classified samples out of all samples in the dataset. It is calculated as Equation 1.


$$\begin{array}{c} \text{Accuracy}=\frac{TP+TN}{TP+TN+FP+FN} \end{array}$$ (1)

**Precision:** Precision evaluates the model’s ability to avoid false positives. It is calculated as Equation 2.


$$\begin{array}{c} \text{Precision (P)}=\frac{TP}{TP+FP} \end{array}$$ (2)

**Recall:** Recall measures the model’s ability to capture all the true positives in a class. It is defined as Equation 3.


$$\begin{array}{c} \text{Recall (R)}=\frac{TP}{TP+FN} \end{array}$$ (3)

**F1-Score:** F1-score is calculated by taking the harmonic mean of precision and recall. It balances the trade-off between the precision and recall as shown in Equation 4.


$$\begin{array}{c} \text{F1-Score}=2\times \frac{P\times R}{P+R} \end{array}$$ (4)

In the above equations, TP, TN, FP, and FN are true positives, true negative, false positive, and false negative, respectively.

## Results and discussion

In this section, we evaluated the proposed model. It comprises each fold results, analysis of F1-score, Accuracy, Loss vs Epoch, comparison of the proposed approach with related work, and baseline methods.

### Fold 1 results

**Fig 2:**
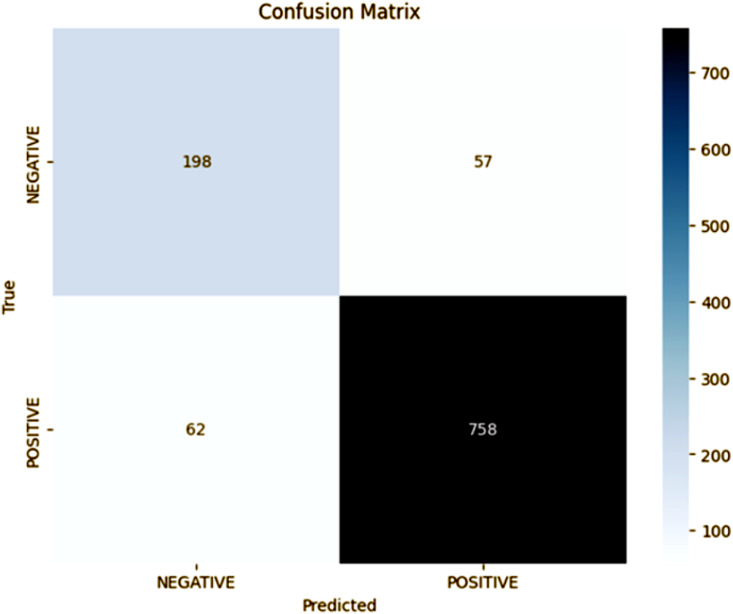
Fold 1 Results.

The results of the confusion matrix for fold 1, as shown in Fig 2, indicate that our proposed model shows good performance in binary classification. The model was accurate 758 times on positive samples (TP) and 198 times on negative samples (TN), giving 91% accuracy. The precision in fold 1 is 93%, which is strongly dependent on the model when it comes to predicting the positives, while the recall of 92% shows correctly labeling the TN despite the existing 62 FN. The F1-score, on the other hand, is computed as 0.92, which is a reinforcement of the balanced approach of the proposed approach with respect to precision and recall, and demonstrates the robustness of the model in dealing with class imbalances. However, the model being slightly susceptible to FP (57 instances) implies that room for improvement could be made in threshold fine-tuning or misclassification redressing.

### Fold 2 results

**Fig 3:**
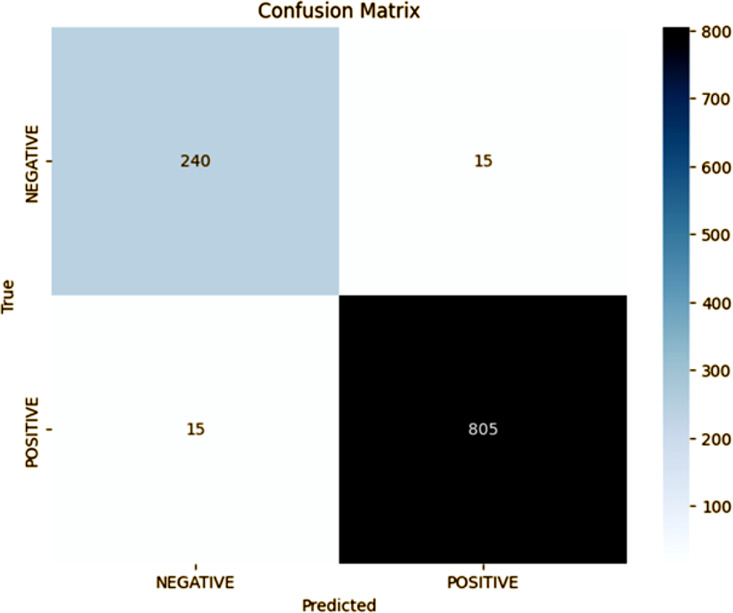
Fold 2 Results.

As shown in Fig 3, Fold 2 results (confusion matrix) show that the model has improved its generalization. In fold 1 the model had an overall accuracy of 91%, with a precision of 93% and recall of 92% for the positive class, giving an F1-score of 92%. However, fold 1 had considerable misclassification rates with 57 FP and 62 FN which indicated the occasional inability of the model to correctly differentiate between the negative and the positive categories. This makes it hard to say how much the performance of the model to identify TP. In contrast, fold 2 got a much higher accuracy of 98%, depicting a substantial improvement of the model’s ability to accurately classify both negative and positive instances. The precision and recall of the positive class rose to 98% with the F1-score improving to 98%, indicating that the proposed model now has an optimal balance between FP and FN rates. In fold 2, the FP and FN have greatly reduced to only 15 instances each, which is much more reliable and precise of a proposed model. This dramatic decrease in misclassifications shows that fold 2 is not only more accurate but also more accurate at generalization across the dataset, which effectively means that the proposed approach substantially decreases the degree of overfitting. These improvements have been attributed to the fact that the model is tuned better and handled class distributions better, and the regularization techniques used in fold 2 have improved and allowed the model to accurately predict both classes. The results from fold 2 show that the model has learned to better differentiate between the subtle differences between the classes and is therefore more appropriate to deploy in high-stakes decision-making applications where precision and recall are both critical.

### Fold 3 results

**Fig 4:**
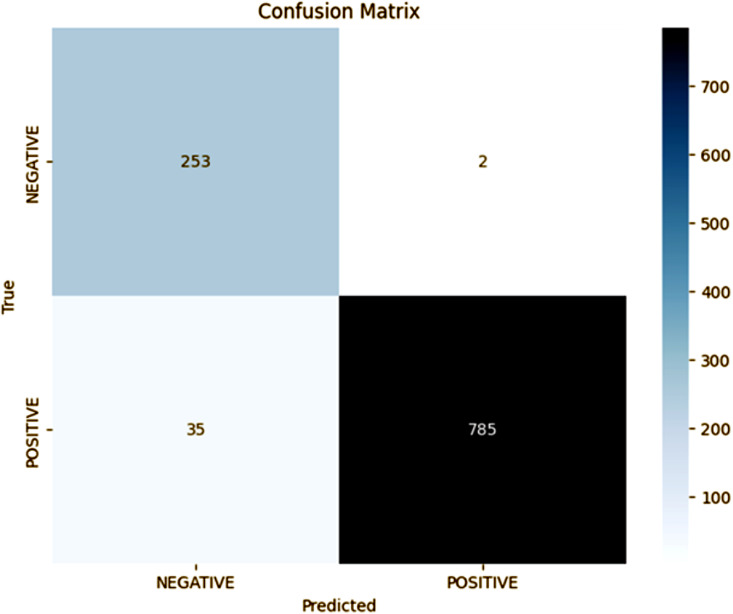
Fold 3 Results.

The fold 3 results of our proposed model, as shown in the confusion matrix in Fig 4, indicate a significant improvement in the classification accuracy and precision. The model is able to correctly detect 785 cases of positive (TP) and 253 cases of negative (TN), which provides an accuracy of approximately 97%. The precision of the positive class is 99%, and the recall is also 96%, which results in an F1-score of 97%. Though the FP and FN reductions to 2 and 35, respectively, further demonstrate the superb capability of demarcating the two classes with minimal misclassification. This fold is a big improvement on the previous folds. Fold 3 shows an improvement in both precision and recall, which means that the model is improving at identifying true positives and showing a very low rate of misclassifications. The results highlight the effectiveness of those adjustments made in fold 3, which further improved the generalization and performance of the model’s reliability and robustness for practical applications.

### Fold 4 results

**Fig 5:**
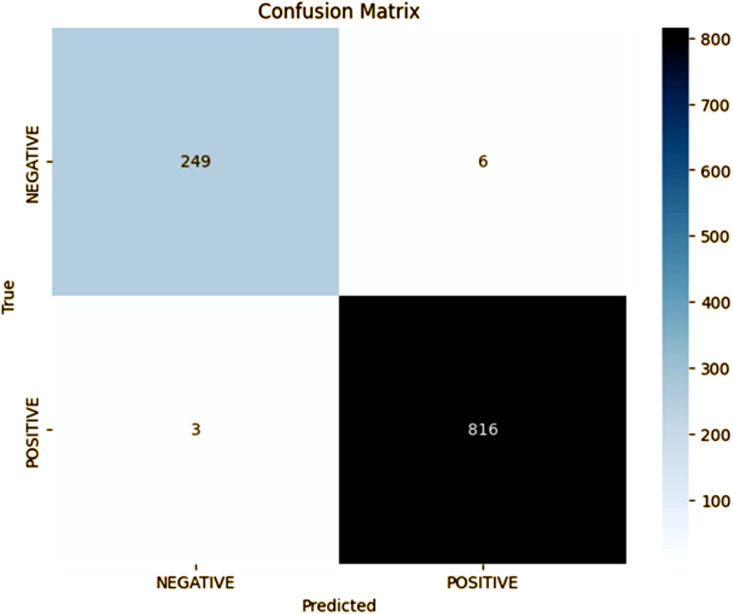
Fold 4 Results.

The results of fold 4, as shown in Fig 5, obtained by our proposed model, further improve the performance obtained in previous folds. The model has been able to predict 816 positive notions (TP) and 249 negative notions (TN) with an accuracy of 98% approximately. The precision for the positive class is 99% and the recall is 99%, and hence the F1 score is calculated as 99%, indicating that there is an excellent balance between precision and recall. The model shows a great reduction in the misclassifications by achieving only 3 FN and 6 FP. These results indicate that fold 4 has a near optimal classification result and a minimal number of errors, further proving the model’s ability of generalization with unseen data. Compared to the previous folds, fold 4 could be seen as an outstanding success in terms of decreasing errors and enhancing both the positive and negative instance detection, which is very reliable in real-life applications where the accuracy with the test data is crucial.

### Fold 5 results

**Fig 6:**
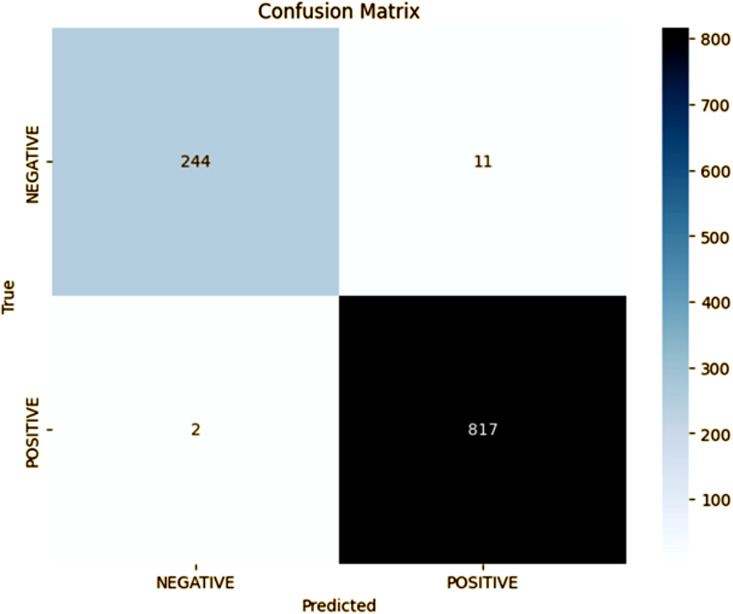
Fold 5 Results.

The fold 5 result further consolidates the proposed model’s outstanding performance on all folds. In fold 5, as shown in the confusion matrix as Fig 6, the model is able to determine 817 positive instances (TP) and 244 negative instances (TN), for an accuracy of 98%. The precision for the positive class is still the same at 99% but the recall is 99%, with the F1-score being 99%. The proposed model shows a minimum number of misclassifications by showing 2 FN and 11 FP. These results are indicative of the model’s ability to make the right distinction between the two classes, with negligible errors, and hence, the robust generalization across the dataset. Compared to previous folds, fold 5 keeps the model’s high reliability and consistency, which indicates the optimization of the hyperparameters of the proposed approach.

To make sure that there was no data leakage in the 5-fold stratified cross-validation, we have strictly separated the train and validation set for each fold, making sure that from the validation set nothing is included in the training process. We also employed stratified sampling to maintain the proportional distribution of classes from one fold to another, to prevent class imbalance. Features were carefully chosen to make sure that there is no implication of future and validation-related information to the model. The model was trained and hyperparameters were fine-tuned considering only the training data of each fold and final evaluations were performed with validation data only. These precautions made sure that the performance metrics reported reflect the true generalization capabilities of the model without any data leakage, as the results are consistent and reliable across all the folds.

### F1-score analysis from Fold 1 to Fold 5 (Fold 0 to Fold 4)

**Fig 7:**
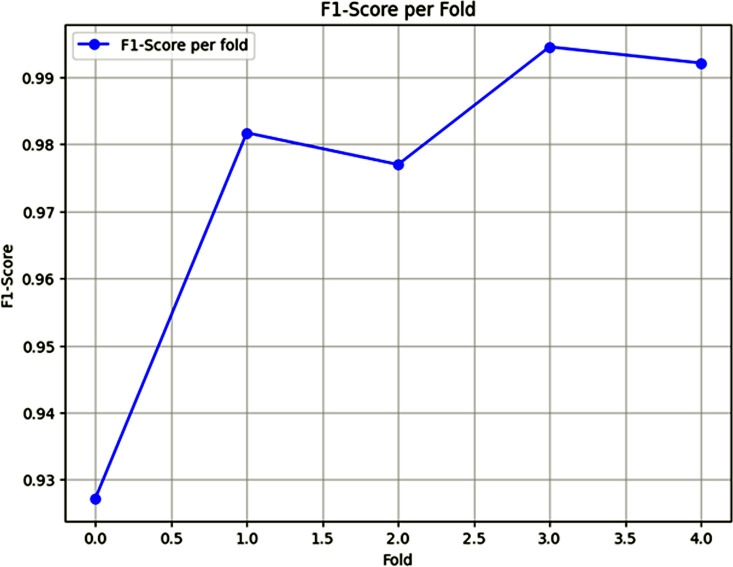
F1-score Per Fold Results.

The F1-score per fold chart, as shown in Fig 7 provides a good picture to see the performance of the model in a number of folds and how the model’s performance improves with time. Starting with an F1-score of 93% in fold 0, as the model moves through additional elapsed model The F1-score has increased by an enormous margin in fold 1–98%, showing that the model has become better as both a discriminator of principle and the discriminator for recall. This increasing tendency persists in the case of fold 2, which has an F1-score of around 99% that depicts the perfect balance of precision and recall. In fold 3, the F1-score remains as high as 99%, showing the consistent performance of the proposed model. However, fold 4 is slightly dipping, showing an F1-score at about 98%, showing some drop in performance from fold 3, but still high effectiveness in general.

### Learning rate vs accuracy

**Fig 8:**
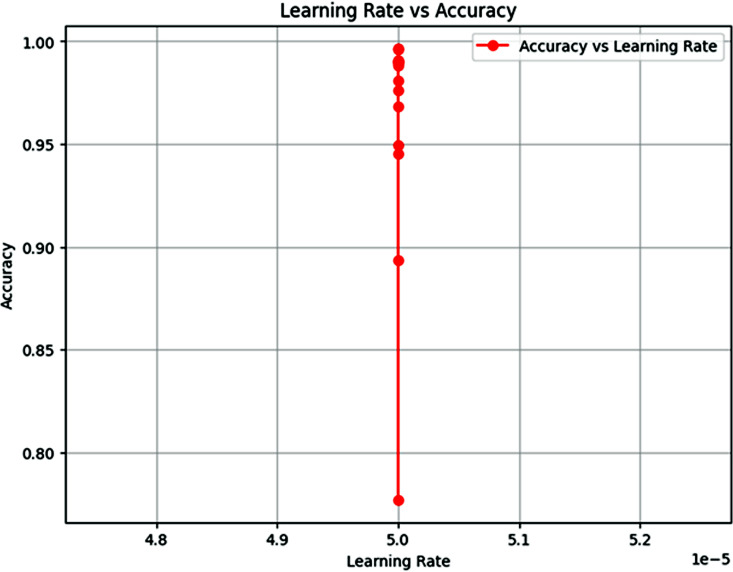
Learning Rate vs Accuracy.

The “Learning Rate vs. Accuracy” plot, as shown in Fig 8 represents a clear and strong relationship between learning rate and accuracy of the proposed model. From the plot, we see that as the learning rate is closer to 5e-5, the model gets to near-perfect accuracy of 1.0, in which there is no significant fluctuation with different trials/iterations. This suggests that the performance of the proposed model is very sensitive to this specific learning rate. The learning rate of 5e-5 may be ideal for the training process of the model. The fact that the amount of accuracy stabilizes at this point implies that any other values of learning rate, either smaller or larger, do not result in such favorable outcomes. This might mean that the training process for the model at a learning rate of 5e-5 is properly balanced, which might be good in terms of avoiding issues such as underfit or overfit, which are more prevalent when using inadequate learning rates. The sharp drop or lack of variation before and after this point indicates that further fine-tuning of the learning rate might lead to sub-optimal performance, and therefore, the learning rate of 5e-5 should be retained for further model training.

### Loss vs Epoch

**Fig 9:**
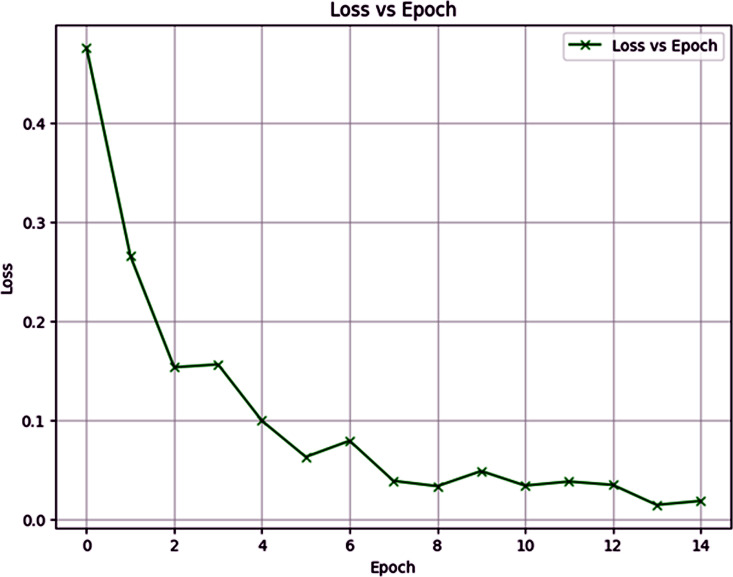
Loss vs Epoch.

The “Loss vs Epoch” plot illustrates in Fig 9 indicates the loss curve of the proposed model for 15 epochs and also how the model’s performance changed as the model was trained. Initially, in the first epoch, the loss is quite high at around 0.45, but there is a steep drop in loss in the second epoch that is suggestive of a significant improvement of the proposed model at an early stage. The loss is still decreasing slowly through the training process and stabilizes around epoch 12. By epoch 14 and the loss is low around 0. This behavior implies that the model learns fast from the data from the starting time of the training process, and the greatest reduction in the loss occurs in the first few epochs. The slowing of the subsequent reduction means that the model has already learned most of the patterns that are in the data, and further training does not yield diminishing returns. The plateau after epoch 12 hints that the proposed model has reached a state of convergence and would not be able to offer any significant improvements through further epochs. This overall loss curve makes the training process look like a well-functioning process.

**Fig 10:**
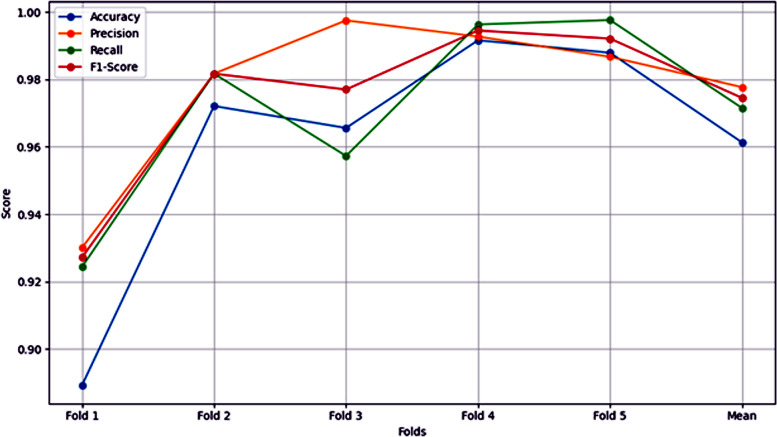
Average Score of Evaluation Metrics.

The average fold score is represented as Table 3 and a graphical representation is shown as Fig 10.

**Table 3:** Average Fold Results.

Fold #	Accuracy (%)	Precision (P) (%)	Recall (R) (%)	F1-Score (%)
1	88.93	93.01	92.44	92.72
2	97.21	98.17	98.17	98.17
3	96.56	99.75	95.73	97.70
4	99.16	99.27	99.63	99.45
5	98.79	98.67	99.76	99.21
**Average**	**96.13**	**97.77**	**97.15**	**97.45**

Table notes: Performance metrics across five stratified folds showing consistency and robustness of the proposed model.

Being a transformer-based architecture, DistilRoBERTa is very good at getting complex contextual relationships at the word level using its attention mechanism, which makes it possible to form representations for it that are sensitive to the underlying semantics in the input text. It processes the input text and generates the contextual embeddings, which represent the meaning of the word in its context (the other words around it) and provide rich semantic representations that are very important for the SA tasks. This is complemented by the BiGRU layer, in which it learns bidirectional sequential dependencies that allow it to learn about the context of every word in the past and future. The two-way nature of BiGRU is very useful as it will look at both the previous and next word, and can learn the dependency that might not be learned by traditional unidirectional RNNs/GRUs, and will lead to a more complete understanding of the sentiment. Moreover, dropout regularization and class weighting are also added to improve the generalization ability of the model. Dropout helps everywhere where overfitting is occurring. Dropout helps by disabling a fraction of neurons randomly at training time, which helps the model not be so dependent on specific features and makes it more robust. Class weighting, on the other hand, is used to solve the problem of class imbalance, where the model could otherwise be biased towards the majority class (negative reviews). By altering the loss function by giving utmost importance to the minority class (positive sentiment), the model to learn from the positive reviews and hence, an improvement is obtained in the recall of the positive reviews without any loss in the precision. The Stratified K-Fold cross-validation technique utilized during training ensures that the model is evaluated in a manner that represents the actual performance of the model with a lot of splits, with a variety of representative distributions of the sentiment classes. This further reduces the potential for overfitting, in that the model is getting tested on different subsets of data, and the results are not too dependent on a certain split in the data. The proposed model shows a technically advanced one in terms of SA by using the language understanding capabilities of DistilRoBERTa and the capacity of modelling sequential dependencies as BiGRU’s model. The combination of dropout, class weighting, and StratifiedKFold cross-validation ensures a good performance and generalization.

The performance of the DistilRoBERTa model, as shown in Table 4, was measured at 4 epochs and the consistent improvement of the model performance in both the training and validation set is noticed. Training loss is reduced from 0.4360 in epoch 1 to 0.0682 in epoch 4 and training accuracy has increased from 75.92% to 97.88%. Validation accuracy was also increased from 90.88% to 95.07% and shows good generalization. The increase in validation F1 score was from 0.9423 to 0.9681, which indicates the balanced precision and recall were also high, with the precision score reaching 0.9571 and the recall score reaching 0.9793 by the 4th epoch. Furthermore, the ROC-AUC score showed that the model had good discrimination ability, which increased from 0.9577 to 0.9854. These results reveal the power of the model, the distributional learning, in learning from the data and making accurate and balanced predictions.

**Table 4:** Performance Evaluation using DistilRoBERTa Model.

Epoch	Train Loss	Train Accuracy	Val Loss	Val Accuracy	Val F1 Score	Val Precision	Val Recall	Val ROC-AUC
1	0.4360	0.7592	0.2648	0.9088	0.9423	0.9112	0.9756	0.9577
2	0.1836	0.9318	0.1939	0.9274	0.9526	0.9492	0.9561	0.9754
3	0.1134	0.9614	0.1718	0.9423	0.9627	0.9491	0.9768	0.9836
4	0.0682	0.9788	0.1854	0.9507	0.9681	0.9571	0.9793	0.9854

The performance of the BiGRU model, as shown in Table 5, was assessed in 4 number of epochs and achieved considerable improvement in training and validation metrics. Training loss went from 0.4759 in epoch 1 to 0.1484 in epoch 4 and training accuracy from 77.64% to 94.46%. Validation accuracy improved from 81.02% to 88.84%, which is a good sign of generalization. The validation F1 score was progressively increasing from 0.8826 to 0.9250, indicating a balanced performance between precision and recall. Validation precision increased from 0.8355 to 0.9487, whereas recall decreased slightly from 0.9354 to 0.9024, and these are good trade-offs in predicting positive classes. Additionally, the validation ROC-AUC score was improved from 0.8074 to 0.9336, which shows the improvement of model’s ability to distinguish the classes as the model progressed over the epochs. These results point to the effectiveness of the BiGRU model to learn from data and make accurate, reliable predictions on a training dataset and a validation dataset.

**Table 5:** Performance Evaluation using BiGRU Model.

Epoch	Train Loss	Train Accuracy	Val Loss	Val Accuracy	Val F1 Score	Val Precision	Val Recall	Val ROC-AUC
1	0.4759	0.7764	0.4760	0.8102	0.8826	0.8355	0.9354	0.8074
2	0.3259	0.8664	0.3379	0.8605	0.9102	0.8941	0.9268	0.8983
3	0.2251	0.9128	0.2960	0.8781	0.9208	0.9126	0.9293	0.9228
4	0.1484	0.9446	0.2919	0.8884	0.9250	0.9487	0.9024	0.9336

As shown in Table 6, the results of the performance evaluation of the models indicate that the Hybrid (DistilRoBERTa + BiGRU) model achieved better performance than both the DistilRoBERTa and BiGRU models in terms of important performance evaluation metrics. With a validation accuracy of 96.13%, the hybrid model is better than 95.07% of DistilRoBERTa and greatly outperforms BiGRU at 88.84%. The hybrid model also attains the validation F1 score of 0.9745, which is higher than the scores of DistilRoBERTa (0.9681) and BiGRU (0.9250) indicating a better balance between precision and recall. Furthermore, the validation precision of the hybrid model is quite high, 97.77%, which is much better than the DistilRoBERTa (95.71%) and the BiGRU (94.87%). Lastly, the validation recall of the hybrid model, with a score of 97.15%, is better than the 90.24% of BiGRU and a bit lower than the 97.93% of DistilRoBERTa, showing a well-rounded capability of identifying both positive and negative cases.

**Table 6:** Performance Results of DistilRoBERTa, BiGRU and DistilRoBERTa-BiGRU.

Model	Val Accuracy	Val F1 Score	Val Precision	Val Recall
DistilRoBERTa	0.9507	0.9681	0.9571	0.9793
BiGRU	0.8884	0.9250	0.9487	0.9024
Hybrid (DistilRoBERTa + BiGRU)	0.9613	0.9745	0.9777	0.9715

**Fig 11:**
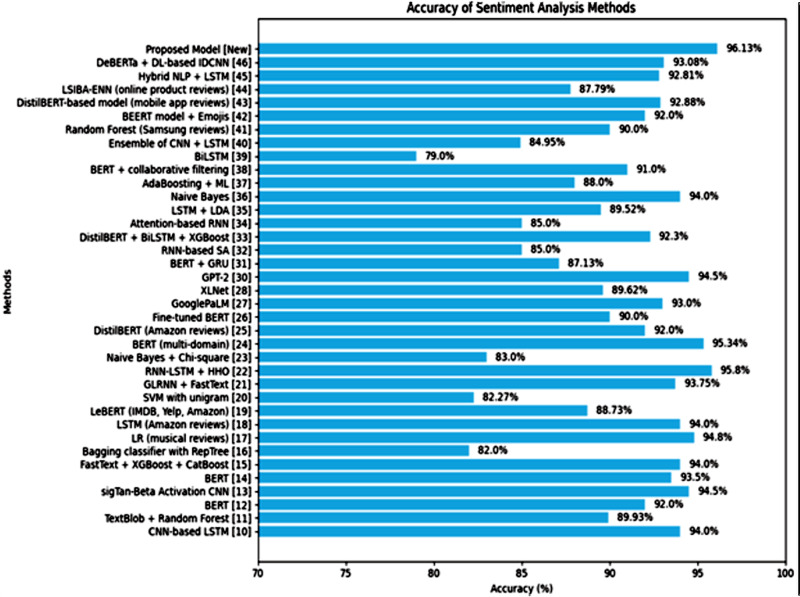
Comparison of Proposed Approach with Related Work.

Our proposed model goes one step beyond by integrating DistilRoBERTa with BiGRU, which outperforms the related work approaches as shown in Table 1 with 96.13% accuracy and Fig 11, while generating deeper insights with the help of appraisal theory and SHAP for interpretability. This makes our approach more robust in dealing with complex linguistic features and improves the scalability to be used on real-world applications like Amazon product reviews and provides a more comprehensive and actionable analysis than traditional models.

### Performance evaluation of proposed approach with related approaches

In comparison to the recent studies in sentiment analysis, our proposed model (DistilRoBERTa + BiGRU) is competitive and unique in a few aspects as shown in Fig 11. While the transformer based and deep learning models like DistilBERT with advanced embeddings [25], GPT based sentiment models [30] and XLNet [28] are shown to be good performers, our proposed model provides a mean fold accuracy of 96.13%, which is higher than these models. Models such as Comprehensive Word Embedding and Feature Design [9] and Deep Learning Predictive Models for Consumer Emotion [10] emphasize more on accuracy or overall classification performance rather than directly learning more semantic aspects of sentiment. Similarly, Comprehending E-commerce Product Reviews [11] is also susceptible to all of these traditional SA pipelines, but no attempt has been made to explain the model interpretation. Our proposed model addresses these limitations by combining contextual embeddings and sequential learning with further improved transparency with SHAP explainability. This allows nuanced insights about emotional tendencies, functional effectiveness and product perceptions to set our proposed model apart not only in its performance but also in its interpretability compared to the other recent works.

### Comparison of proposed model over existing approaches

**Fig 12:**
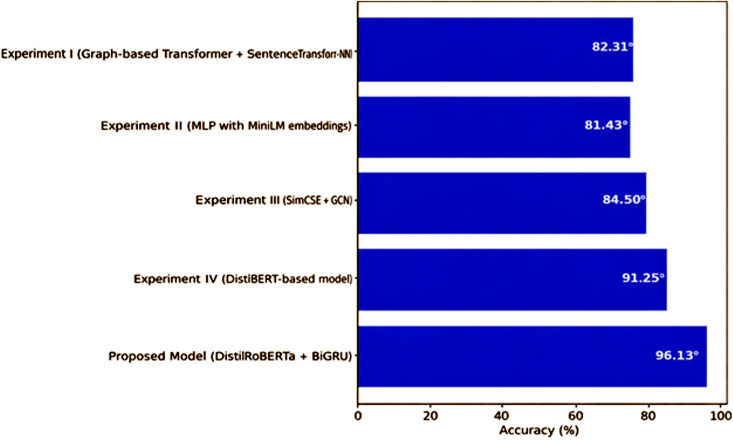
Comparison of Proposed Model with Existing Approaches.

As shown in Fig 12, the proposed model (DistilRoBERTa + BiGRU) outperforms all the existing approaches, as it has the highest accuracy of 96.13%, which is significantly higher than the other approaches that we have tested during this research study. Compared with Experiment I (Graph-based Transformer + SentenceTransformer + k-NN) 82.31%, Experiment II (MLP with MiniLM embeddings) 81.43%, Experiment III (SimCSE + GCN) 84.50%, and Experiment IV (DistilBERT-based model) 91.25%, the proposed model shows a significant superiority. This outstanding performance indicates the performance of the hybrid approach in which DistilRoBERTa’s strong natural language understanding capabilities and BiGRU layer’s sequential modeling capability are combined, taking care of both superior context understanding and sequential dependency modeling in the SA tasks.

## Conclusion and future work

This study proves that a hybrid DL strategy is effective in the analysis of the Amazon product performance based on a SA. With the combination of DistilRoBERTa and BiGRU, the model reveals contextual as well as sequential product review dependencies, in contrast to the conventional polarity-related approaches when the complex linguistic markers, like complexity and usability, are considered in line with the appraisal theory. The model attains great results, and its average fold accuracy is 96.13, which is better than the current methods. Moreover, when XAI is implemented with the help of SHAP, the model can be made more interpretable, which allows for identifying emotional and functional implications of user feedback. As future research, the issue of SA in low-resource languages should be resolved. To model such languages, one possibility can be to use techniques such as fine-tuning on data with many languages, transfer learning, or the generation of synthetic data using data augmentation. Further, cross-lingual embeddings and multilingual models can be combined to help in the capture of sentiment, involvedness and practicality under different linguistic environments. It would make sure that the model becomes scalable and applicable to the global markets, especially in the areas where low-resource languages are common, and that further streamlines the product development strategies and ultimate decision-making process

## Supporting information

S1 File (CSV)
